# Effect of fluoroscopic X‐ray beam spectrum on air‐kerma measurement accuracy: implications for establishing correction coefficients on interventional fluoroscopes with KAP meters

**DOI:** 10.1120/jacmp.v17i3.6092

**Published:** 2016-05-08

**Authors:** Kevin A. Wunderle, Joseph T. Rakowski, Frank F. Dong

**Affiliations:** ^1^ Department of Radiology Cleveland Clinic Cleveland OH USA; ^2^ Department of Radiation Oncology Wayne State University School of Medicine Detroit MI USA

**Keywords:** fluoroscope, DAP meter, KAP meter, diamentor, air kerma

## Abstract

The first goal of this study was to investigate the accuracy of the displayed reference plane air kerma ($K_{a,r}$) or air kerma‐area product ($P_{k,a}$) over a broad spectrum of X‐ray beam qualities on clinically used interventional fluoroscopes incorporating air kerma‐area product meters (KAP meters) to measure X‐ray output. The second goal was to investigate the accuracy of a correction coefficient (CC) determined at a single beam quality and applied to the measured $K_{a,r}$ over a broad spectrum of beam qualities. Eleven state‐of‐the‐art interventional fluoroscopes were evaluated, consisting of eight Siemens Artis zee and Artis Q systems and three Philips Allura FD systems. A separate calibrated 60 cc ionization chamber (external chamber) was used to determine the accuracy of the KAP meter over a broad range of clinically used beam qualities. For typical adult beam qualities, applying a single CC determined at 100 kVp with copper (Cu) in the beam resulted in a deviation of <5% due to beam quality variation. This result indicates that applying a CC determined using The American Association of Physicists in Medicine Task Group 190 protocol or a similar protocol provides very good accuracy as compared to the allowed ±35% deviation of the KAP meter in this limited beam quality range. For interventional fluoroscopes dedicated to or routinely used to perform pediatric interventions, using a CC established with a low kVp (∼55−60 kVp) and large amount of Cu filtration (∼0.6−0.9 mm) may result in greater accuracy as compared to using the 100 kVp values. KAP meter responses indicate that fluoroscope vendors are likely normalizing or otherwise influencing the KAP meter output data. Although this may provide improved accuracy in some instances, there is the potential for large discrete errors to occur, and these errors may be difficult to identify.

PACS number(s): 87.59.C‐, 87.59.cf, 87.53.Bn

## I. INTRODUCTION

The allowable tolerance for the displayed reference plane air kerma ($K_{a,r}$) or air kerma‐area product ($P_{k,a}$) on interventional C‐arm fluoroscopes is ±35% per International Electrotechnical Commission (IEC) and the United States Food and Drug Administration (USFDA) requirements.^(1)^ Large deviations in the accuracy of the displayed air kerma are therefore possible, even for properly functioning calibrated fluoroscopic systems. Inaccuracies of this magnitude are untenable for the purposes of estimating patient radiation dose or establishing and comparing clinical procedure reference dose levels, as suggested by the National Council on Radiation Protection and Measurements in Report 168.^2^, ^3^ The International Commission on Radiation Units and Measurements (ICRU) has recommended that uncertainty should be within 7% for radiation dose quantities in diagnostic imaging, a seemingly impossible task without correcting for the allowed inaccuracy of the displayed $K_{a,r}$.^(4)^

The accuracy and variability of stand‐alone air kerma‐area product (KAP) meters have been investigated by various groups since these meters came into common use a couple of decades ago.^5^, ^6^, ^7^, ^8^ Toroi et al.^(8)^ investigated the response of various standalone KAP meters over a range of X‐ray beam qualities, varying both the kVp and filtration. The results indicated that the correction coefficients (CCs) decrease as a function of increasing kVp from 40 kVp through 90 kVp; beyond that, the coefficient response was generally flat. The coefficients also decreased with increasing beam hardness (larger amounts of filtration) at a given kVp. These reports suggest that the standalone KAP meters evaluated generally have a higher response (lower calibration coefficient) as the beam quality is increased. However, these investigations were performed on standalone KAP meters that functioned independently of the fluoroscopic systems. Over the last decade, KAP meters have been integrated into the fluoroscopic assembly, with their measurements displayed alongside other fluoroscopic technical parameters. How these measurements are integrated, and the effect of no longer having a fully independent measuring system, have not been investigated.

Additionally, the American Association of Physicists in Medicine (AAPM) established a Task Group (TG‐190) to define a protocol for determining and implementing CCs for a wide variety of X‐ray equipment, including C‐arm fluoroscopes used for interventional procedures. The TG‐190 report, titled “Accuracy and calibration of integrated radiation output indicators in diagnostic radiology: a report of the AAPM Imaging Physics Committee Task Group 190,”^(9)^ provides standardized protocols including system geometry and recommended fluoroscope settings for determining a CC. Specifically for C‐arm fluoroscopes used for interventional procedures, TG‐190 recommends:

1. Free in‐air geometry
2. Measurement by an external dosimeter situated at the isocenter of the C‐arm
3. Testing of acquisition and fluoroscopy modes within a routine clinical examination set
4. 100±10 kVp as the reference kVp
5. Maximum source‐to‐image receptor distance (SID)

For the present study, there were two primary purposes. The first was to determine the accuracy of the displayed $K_{a,r}$ or $P_{k,a}$ over a broad spectrum of X‐ray beam qualities on clinically used interventional fluoroscopes with integrated KAP meters to measure X‐ray output. The second purpose was to investigate the accuracy of using a CC determined at a single beam quality and applying that CC to a broad spectrum of beam qualities, as suggested by TG‐190.

## II. MATERIALS AND METHODS

**Figure 1:**
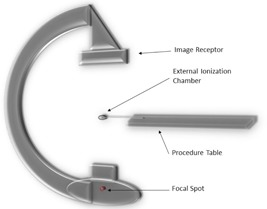
Setup for C‐arm and external ionization chamber.

This investigation was limited to interventional C‐arm fluoroscopes using KAP meters (also known as DAP meters, AKAP meters or diamentors) to measure $P_{k,a}$ and/or $K_{a,r}$. Eleven state‐of‐the‐art interventional fluoroscopes were evaluated, including Siemens (Siemens Healthcare, Erlangen, Germany) Artis zee and Artis Q systems and Philips (Philips, Best, Netherlands) Allura FD systems. All units evaluated were fixed C‐arm type fluoroscopes with flat‐panel digital image receptors. A Radcal (Radcal Corporation, Monrovia, CA) Accupro dosimeter with calibrated Radcal 10×6−60 (60 cc) ionization chamber (external chamber) was used to evaluate the accuracy of the KAP meter. The C‐arm was positioned with the X‐ray tube near the floor and the image receptor above, the typical orientation for posteroanterior projections with a supine patient. No objects (including the procedure table and pad) were in the path of the X‐ray beam; the IEC standard for the indicated air kerma or air kerma‐area product of these fluoroscopes specifies free in‐air geometry without the procedure table present. The external chamber was mounted off the end of the procedure table, at or near the isocenter of the C‐arm, with an SID of 100 cm or greater (Fig. 1). A lead plate was used to cover and protect the image receptor during irradiation, but this plate was located sufficiently far away from the external chamber to prevent scatter radiation from affecting the measurements. A radiopaque ruler was used to measure the linear dimensions of a collimated square X‐ray field, which was set to approximately 10×10 cm in the plane of the external chamber.

On each fluoroscopic system, three measurements were made at each available spectral filter (copper [Cu]) thickness for kVps ranging from 55 through 125 in 10 kVp increments and at 100 kVp (the TG‐190 reference kVp). To accomplish this, the vendor service mode (Siemens) or service assistance (Philips) was required to set fixed radiographic techniques. The $P_{k,a}$ measured by the KAP meter and the incident air kerma ($K_{a,i}$) measured by the external chamber were recorded for each exposure. The $K_{a,i}$ measurements from the external chamber were multiplied by the X‐ray beam area, yielding a $P_{k,a,\text{ex}}$ that was divided by the $P_{k,a}$ measured by the KAP meter, providing a correction coefficient (CC):

(1) $$CC=\frac{K_{a,i}*A}{P_{k,a}}$$

where $K_{a,i}$ is the incident air kerma measured by the external ion chamber, *A* is the area of the X‐ray beam in the plane of the external ion chamber, and $P_{k,a}$ is the air kerma‐area product reported by the fluoroscope.

In addition to CCs, normalized correction coefficients (NCCs) were determined by taking the CCs at each beam quality and normalizing them to one of the CCs determined at 100 kVp (the TG‐190 reference kVp) with different filtration thicknesses:

(2) $$NCC_{100,0}(i,j)=\frac{CC_{i,j}}{CC_{100,0}}$$

(3) $$NCC_{100,0.1}(i,j)=\frac{CC_{i,j}}{CC_{100,0.1}}$$

(4) $$NCC_{100,0.9}(i,j)=\frac{CC_{i,j}}{CC_{100,0.9}}$$

where *i* and *j* represent the kVp and Cu filtration thickness (mm), respectively. The NCCs provide an estimated deviation for using the 100 kVp CC instead of the CC at each specific beam quality.

## III. RESULTS

**Figure 2:**
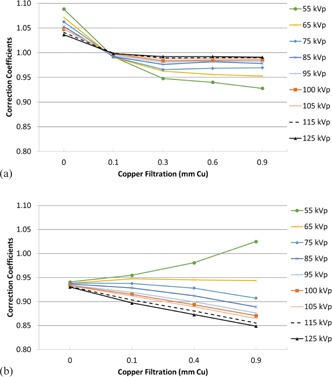
Averaged correction coefficients for (a) the Siemens units and (b) the Philips units.

**Figure 3:**
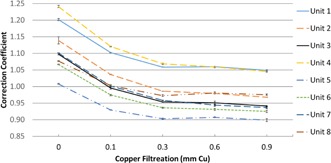
Correction coefficients at 55 kVp for the Siemens units.

Figures 2(a) and 2(b) show the average CCs for each of the vendors at each beam quality. The reported values should not be used for any quantitative or clinical application; they are provided strictly to portray the trend of the CCs at each kVp as filtration is increased. There was significant intersystem variability in the CCs; Fig. 3 shows this variability for the eight Siemens systems at 55 kVp. However, as illustrated by the error bars in Fig. 3, the intrasystem variability of the three measurements made at each beam quality was very small, and the trend of CCs for each vendor with increasing beam quality was consistent. Although not shown, the Philips systems exhibited similar inter‐ and intrasystem variability.

**Figure 4:**
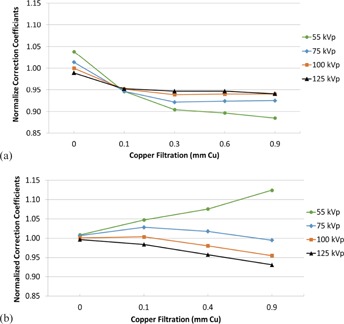
Normalized correction coefficients for the Siemens units, normalized to the correction coefficient at 100 kVp without filtration (a); same conditions for (b) the Philips units.

**Figure 5:**
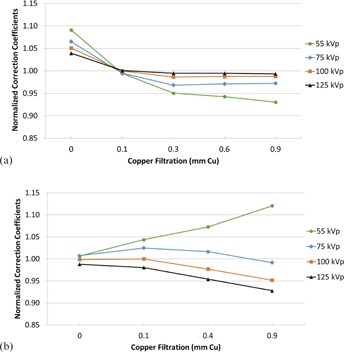
Normalized correction coefficients for the Siemens units, normalized to the correction coefficient at 100 kVp with 0.1 mm of filtration (a); the same conditions for (b) the Philips units.

**Figure 6:**
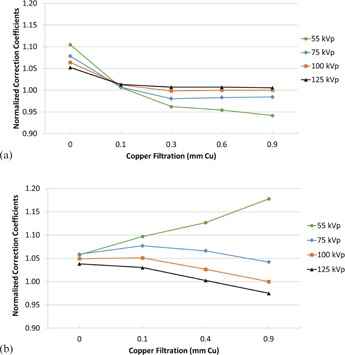
Normalized correction coefficients for the Siemens units, normalized to the correction coefficient at 100 kVp with 0.9 mm of filtration (a); the same for (b) the Philips units.

Figures 4(a) and 4(b) illustrate the CC at each beam quality normalized to the CC at 100 kVp without additional filtration (${\text{NCC}}_{100,0}$[i,j]) for Siemens and Philips, respectively. Figures 5(a) and 5(b) illustrate the CC at each beam quality normalized to the CC at 100 kVp with 0.1 mm of additional filtration (${\text{NCC}}_{100,0.1}$[i,J]). Figures 6(a) and 6(b) illustrate the CC at each beam quality normalized to the CC at 100 kVp with 0.9 mm of Cu filtration (${\text{NCC}}_{100,0.9}$[i,j]).

## IV. DISCUSSION AND CONCLUSION

**Figure 7:**
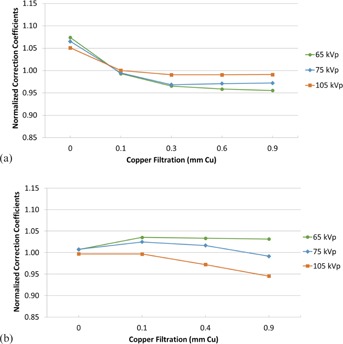
Normalized correction coefficients for the Siemens units (a) over a typical beam quality range for adult fluoroscopic imaging, normalized to the correction coefficient at 100 kVp with 0.1 mm of filtration; normalized correction coefficients for the Philips units (b) over a typical beam quality range for adult fluoroscopic imaging, normalized to the correction coefficient at 100 kVp with 0.1 mm of filtration.

A primary goal of this study was to investigate the accuracy of applying a CC determined at a single beam quality, such as that established by AAPM TG‐190, to a broad spectrum of beam qualities on interventional C‐arm fluoroscopes. In this regard, two primary conclusions can be drawn from the data acquired. First, for typical adult beam qualities, applying a single CC determined at 100 kVp with Cu in the beam results in a deviation of less than 5% due to beam quality variation. Figures 7(a) and 7(b) illustrate the ${\text{NCC}}_{100,0.1}$(i,j) for a typical range of beam qualities used during adult fluoroscopic imaging (65‐105 kVp; 0.1‐0.6 mm of Cu). This indicates that applying a CC determined using the TG‐190 (or similar) protocol provides very good accuracy as compared to the allowed ±35% deviation of the KAP meter in this limited beam quality range typically used for adult imaging.

Secondly, for pediatric interventions in children without an adult body habitus, typical beam qualities incorporate low kVp values (∼55 kVp) and large amounts of spectral filtration (0.4‐0.9 mm of Cu). Figures 4(a) and 4(b) indicate that for these beam qualities, the ${\text{NCC}}_{100,0.1}$(i,j) deviates an average of 7% and 12% for the Siemens and Philips units, respectively. For interventional fluoroscopes dedicated to or routinely performing pediatric interventions, using a CC established with a low kVp (∼55−60 kVp) and large amount of Cu filtration (∼0.6−0.9 mm) may result in better accuracy as compared to using a CC determined at 100 kVp.

The stark difference in CCs for the two vendors (Figs. 1(a) and 1(b)) is surprising, as the KAP meters used by both Siemens and Philips for the fluoroscopes evaluated were manufactured by the same vendor, PTW (PTW‐Freiburg GmbH, Freiburg, Germany). In fact, the change in CCs with increasing beam quality (for beam qualities incorporating Cu) was inverse between Philips and Siemens. For the Philips systems, the CCs decreased with increasing beam quality (similar to previous reports),^(8)^ whereas for the Siemens systems, the CCs increased with increasing beam quality. Both the Philips and Siemens systems exhibited a convergence of the CCs at a given beam quality, something not reported by others who have investigated standalone KAP meter responses.^5^, ^6^, ^7^, ^8^ These results indicate that the fluoroscope vendors are likely normalizing or otherwise influencing the KAP meter output data. Even if the purpose is to increase the accuracy of the reported radiation quantities, modification of the raw KAP meter measurements presents opportunities for large isolated deviations, which could easily go undetected. The authors have seen, in unrelated fluoroscope testing, a fluoroscope on which an individual protocol setting resulted in a deviation of approximately 80% compared to all other settings (deviation only occurred during a 30 pps acquisition, and no other setting produced a similar magnitude of error); this appeared to be a lookup‐table (LUT)‐based error and was only discovered by chance. Modification of the KAP meter data by the fluoroscope vendor allows for potentially large discrete errors to occur, and these errors may be nearly impossible for the end user or clinical physicist to identify. Vendors should clearly state what correction factors are being applied to the KAP meter (or provide access to view the LUTs) so that erroneous values may be identified more readily.

The use of a single CC across a wide spectrum of beam qualities can be successfully achieved, greatly improving the accuracy of the displayed $K_{a,r}$ as compared to the ±35% deviation allowed by regulations. The CCs are necessary for calculating peak skin dose estimates or for determining and comparing procedure reference dose levels. Without accounting for these deviations, it should be assumed that there is significant variability in the procedure $K_{a,r}$ or $P_{k,a}$. The AAPM TG‐190 protocol should be widely adopted and included as part of all fluoroscope acceptance and annual testing, with the resultant CCs included in all fluoroscopic testing reports.

## COPYRIGHT

This work is licensed under a Creative Commons Attribution 4.0 International License.

## References

[1] International Electrotechnical Commission. Medical electrical equipment — Part 2‐43: Particular requirements for the basic safety and essential performance of X‐ray equipment for interventional procedures. IEC 60601‐2‐43. Geneva: IEC; 2010.

[2] Jones AK and Pasciak AS . Calculating the peak skin dose resulting from fluoroscopically guided interventions. Part I: Methods. J Appl Clin Med Phys. 2011;12(4):3670. [erratum in J Appl Clin Med Phys. 2014;15(4):402]. 28297242 10.1120/jacmp.v15i4.4986 PMC6458842

[3] National Council on Radiation Protection and Measurements. NCRP Report No. 168 — Radiation dose management for fluoroscopically‐guided interventional medical procedures. Bethesda, MD: NCRP; 2010.

[4] Patient dosimetry for × rays used in medical imaging [committee report]. J ICRU. 2005;5(2):iv–vi. 24170885 10.1093/jicru/ndi018

[5] Larsson JP , Persliden J , Carlsson GA . Ionization chambers for measuring air kerma integrated over beam area. Deviations in calibration values using simplified calibration methods. Phys Med Biol. 1998;43(3):599–607. 9533138 10.1088/0031-9155/43/3/011

[6] Malusek A , Larsson JP , Carlsson GA . Monte Carlo study of the dependence of the KAP‐meter calibration coefficient on beam aperture, x‐ray tube voltage and reference plane. Phys Med Biol. 2007;52(4):1157–70. 17264377 10.1088/0031-9155/52/4/020

[7] Toroi P , Komppa T , Kosunen A . A tandem calibration method for kerma‐area product meters. Phys Med Biol. 2008;53(18):4941–58. 18711243 10.1088/0031-9155/53/18/006

[8] Toroi P , Komppa T , Kosunen A , Tapiovaara M . Effects of radiation quality on the calibration of kerma‐area product meters in x‐ray beams. Phys Med Biol. 2008;53(18):5207–21. 18728309 10.1088/0031-9155/53/18/024

[9] Lin PJ , Scheuler BA , Balter S , et al. Accuracy and calibration of integrated radiation output indicators in diagnostic radiology: a report of the AAPM Imaging Physics Committee Task Group 190. Med Phys. 2015;42(12):6815. 26632039 10.1118/1.4934831

